# Early generation dynamic and static proton arc treatment planning algorithms assessment in oropharyngeal cancer patients

**DOI:** 10.1002/mp.17916

**Published:** 2025-07-15

**Authors:** Bas Adriaan De Jong, Lewei Zhao, Peilin Liu, Viktor Wase, Otte Marthin, Johan Sundström, Gang Liu, Rohan Deroniyagala, Xiaoqiang Li, Xiaoda Cong, Guillaume Janssens, Erik Engwall, Erik, W. Korevaar, Xuanfeng Ding, Johannes Albertus Langendijk, Stefan Both

**Affiliations:** ^1^ Department of Radiation Oncology University Medical Center Groningen University of Groningen Groningen The Netherlands; ^2^ Department of Radiation Oncology Stanford University Palo Alto California USA; ^3^ Department of Radiation Oncology Corewell Health System Rose Cancer Center Royal Oak Michigan USA; ^4^ Research and Development RaySearch Laboratories AB Stockholm Sweden; ^5^ Research and Development Ion Beam Applications SA Louvain‐la‐Neuve Belgium

**Keywords:** delivery time, dynamic and Static PAT, normal tissue complication probability (NTCP), oropharyngeal cancer, proton treatment, robustness

## Abstract

**Background:**

Compared with intensity modulated proton therapy (IMPT), proton arc treatment (PAT) employs an increased number of gantry angles, potentially reducing healthy tissues doses, especially for complex target geometries found in oropharyngeal cancer (OPC) treatment. PAT plans can be optimized with algorithms, based either on “static” gantry position or “dynamic” gantry movement during dose delivery. Recent results have shown target coverage may suffer more from inter‐fraction patient anatomical‐ and setup changes In PAT than IMPT.

**Purpose:**

We assessed if PAT plans generated with one static and two dynamic PAT planning algorithms can improve expected plan toxicity, delivery time, and inter‐fraction robustness compared to clinical volumetric modulated arc therapy (VMAT) and IMPT plans for OPC patients.

**Methods:**

Six OPC patients were included that qualified for proton therapy based on model‐based selection, with IMPT plans superior to the VMAT plans in terms of toxicities. Static PAT plans were produced using an energy layer filtration (ELF) algorithm, and dynamic PAT plans were produced with two different methods: (1) Spot scanning Proton Arc (SPArc), and (2) Early Layer and Spot Assignment (ELSA). Two sets of PAT plans with about 360 or 240 energy layers and an additional ELF plan employing anterior oblique range shifted fields were produced. All proton plans were robustly optimized. Expected plan toxicity was determined using normal tissue complication probability (NTCP) models for dysphagia and xerostomia. A delivery time model was calibrated using experimental machine log‐files and gantry dynamics from an IBA Proteus PLUS system (IBA Ltd, Belgium). Inter‐fraction robustness was evaluated on a fraction‐wise (on weekly repeated CT) and course‐wise (accumulated over all weekly repeated CTs) basis.

**Results:**

All PAT strategies showed significantly (*p*‐values < 0.05) reduced NTCPs for dysphagia and xerostomia grade ≥ 2 compared to IMPT and VMAT. Relative to VMAT, NTCP for xerostomia reduced by on average 4.0% in IMPT plans, 9.8% in ELF‐, 9.6% in SPArc‐, and 8.7% in ELSA PAT plans with 360 energy layers, and NTCP for dysphagia reduced by on average 9.6% in IMPT plans, 13.1% in ELF, 12.9% in SPArc, and 12.3% in ELSA PAT plans. Average clinical IMPT delivery time was 11.4 ± 2.1 min, while dynamic PAT delivery time was on average 7.6 ± 0.5 min for ELSA and 8.2 ± 0.5 min for SPArc plans with 360 energy layers and auto beam sequencing delivery for 360 energy layer 30 beam angle ELF plans was 9.9 ± 1.0 min. Reduction of energy layers to 240 resulted in limited NTCP increase, globally < 1%, and reduced delivery time by up to 3 min, where changes in delivery time and NTCP were largest in ELF plans. Fraction‐wise target coverage was worse in the PAT plans compared to IMPT; however the course‐wise coverage showed to be less impacted by inter‐fraction changes. The addition of anterior oblique fields with range shifters markedly improved fraction‐ and course‐wise target coverage for the ELF plans.

**Conclusions:**

The tested static and dynamic PAT planning strategies showed similar significant reductions in NTCP compared to IMPT and VMAT. Estimated PAT delivery times were shorter compared to times for current delivery procedures of clinical IMPT plans. Dynamic PAT delivery is faster than static PAT. Fraction‐wise robustness suffered more than course‐wise robustness due to anatomical changes found in repeated CTs. Range shifters can be employed to improve PAT plan robustness.

## 1 INTRODUCTION

Oropharyngeal cancer (OPC) is one of the more common subtypes of head and neck cancer and radiation therapy plays an important role in the treatment of OPC patients. Radiotherapy can be employed with curative or palliative intent either as a stand‐alone treatment or in combination with surgery and chemotherapy in a neoadjuvant, concurrent, or adjuvant setting.^1^ In the curative setting the highest radiation dose is delivered to both the primary tumor site and lymph node metastasis, while an elective dose is delivered to the regional lymph nodes to reduce the risk of local and locoregional recurrence. The treatment target volume can be quite extensive, oddly shaped, and boarder a large number of organs at risk (OAR).

In general, the aim of radiotherapy is to deliver a therapeutic dose to the target, while the dose to healthy surrounding tissues is kept as low as possible to minimize treatment side effects which have a negative impact on quality of life.^2^, ^3^ Currently, OPC patients in the Netherlands are treated with volumetric modulated arc therapy (VMAT) or intensity modulated proton therapy (IMPT). Which of these treatments they receive depends on the expected benefit in terms of decreased NTCP IMPT can offer over VMAT using model‐based selection together with patient preferences.^4^, ^5^ IMPT in head and neck patients typically uses 4–6 gantry angles, from directions chosen for good access to the target avoiding shooting through regions with increased patient positioning variation such as the shoulders and skinfolds in the neck. Typically, in IMPT sets of 30 proton energies called energy layers, containing on average 50 spots are employed per field, to cover the target both distally and laterally, however these numbers can vary widely depending on target geometry.

In PAT dose is delivered from a larger number of gantry angles, which yields more flexibility in treatment plan optimization, resulting in further OAR dose and NTCP reductions compared to IMPT^6^, ^7^, ^8^The goal in PAT treatment planning, is to find the optimal distribution of energy layers and spots over the gantry angles, to deliver the treatment dose in a time‐efficient way, with minimal OAR dose. Furthermore, plan optimization time has to be reasonably low to optimize the logistics of treatment preparation.^9^

Different PAT techniques have been proposed, in 1997 the potential benefits of PAT for chest wall irradiations were studied using a rotational passive scattering technique.^10^ Furthermore, distal edge tracking techniques were considered, in which only spots on the distal edge of the target were used and the number of static beams increased to typically 12–24. However, these strategies lacked robustness against setup uncertainties in head and neck patients.^11^ In 2013 Seco et al. used 10° spacing of passive scatter‐ and IMPT beams, and found improved robustness and conformality of the Arc strategies for non‐small cell lung cancer patients^12^ In 2015 Proton, Modulated Arc Therapy (PMAT) was proposed in which a single proton energy was selected for a section of the gantry arc trajectory, allowing for faster “dynamic delivery” with continuous gantry rotation during dose delivery. PMAT had potential to focus linear energy transfer (LET) in the target, resulting in improved biologically effective dose distributions, however robust optimization was not used.^13^, ^14^

In 2016, Spot scanning proton arc therapy (SPArc) was introduced, in which 249 energy layers in a 36‐field IMPT plan were re‐distributed over 84 gantry angles and robustly optimized, resulting in delivery efficient plans with significantly reduced integral‐ and OAR dose and NTCP for bilateral head and neck cancer patients compared to IMPT^15^ However, optimization times were still rather long.^16^, ^17^ Energy layer sequencing was later added to the SPArc methodology to reduce delivery time caused by the asymmetry in proton energy up‐ and down switching time on current treatment machines.^18^ In 2019, the feasibility of SPArc plan delivery was proven using a prototype phantom irradiation.^16^ In 2022, Early energy layer selection and spot assignment (ELSA) was introduced in which a single energy layer per gantry angle is selected in less than 6 min prior to robust spot weight optimization. ELSA plans employing about 180 energy layers were suitable for dynamic delivery and provided reduced integral dose compared to IMPT for prostate cancer patients.^19^ For static arcs, an energy layer filtration (ELF) algorithm has been proposed to iteratively reduce the number of energy layers in an IMPT multi‐field plan.^7^, ^8^, ^20^ Static arc plans employing 360 energy layers distributed over 30 gantry angles also significantly reduced integral‐ and OAR dose and NTCP in OPC patients.^7^, ^8^ Automatic beam sequencing delivery or an arc partitioning delivery strategy could reduce delivery time for ELF plans to become clinically acceptable on current hardware.^7^, ^20^

A recent review article on particle arc therapy, mentioned that robustness of target coverage to patient positioning and inter‐fractional patient changes may be more challenging for PAT compared to IMPT, due to interlaced spot maps coming from a multiple of directions, compared to a set of stacked energy layers from a few directions in IMPT^20^According to the review, extensive additional studies are warranted to address the inter‐fractional robustness of PAT, especially in head and neck cancer patients. In previous head and neck cancer PAT planning studies, typically no strategies were used to improve treatment robustness against patient density and setup uncertainties beyond robust optimization.^7^, ^17^, ^21^ However, it is known that the setup uncertainty for the shoulders and skinfolds in the neck may be larger than the typical 3 mm robustness margin.^22^ Furthermore, the patient may change in between fractions caused by for example weight loss or tumor shrinkage, with potential impact on the delivered dose distribution. Early results have shown the impact of patient changes found in repeated imaging along the treatment has a larger impact on PAT dose distributions compared with IMPT.^20^, ^23^, ^24^

Multiple PAT optimization strategies have shown potential to reduce NTCP and OAR dose, while providing robust target coverage on the planning CT, with clinically feasible delivery time. Information on the robustness of PAT to inter‐fractional patient changes is still scarce in literature.^24^ A comparison of the performance of existing PAT methodologies could help to decide what strategy to go for upon clinical implementation of PAT treatment. We assessed if PAT plans generated using the SPArc, ELSA end ELF methodologies can reduce NTCP, delivery and optimization time and improve inter‐fraction robustness compared to clinical VMAT and IMPT plans for OPC patients.

## 2 MATERIALS AND METHODS

### 2.1 Patient cohort and clinical treatment planning

Six OPC patients treated with IMPT, for whom no adaptive replanning was performed, were used in this study. A radiation dose of 7000 cGy was prescribed for the therapeutic clinical target volume containing the primary tumor and pathological lymph nodes (CTV 7000), 5425 cGy was prescribed for the prophylactic clinical target volume containing the elective lymph node area (CTV 5425). During treatment, a 5‐point mask was used for immobilization, and orthogonal x‐ray imaging was employed to correct patient posture. Subsequently, cone beam CT (CBCT) images were acquired and rigidly matched to the planning CT. The resulting translation was applied to the patient using a 6‐D robotic table.

For all patients, clinical VMAT and IMPT plans were produced in a clinical version of RayStation (version 9A, RaySearch Laboratories, Stockholm, Sweden) treatment planning system (TPS), for the purpose of model‐based selection.^4^ The treatment plans had to adhere to clinical goals regarding minimal target coverage to ensure adequate treatment and maximum dose to avoid excessive toxicity, defined in more detail in Appendix A, Table A1. All proton plans in this study were robustly optimized using 3 mm setup and 3% density uncertainty settings in a composite worst‐case (minimax) optimization using a beam model for an IBA Proteus PLUS (IBA Ltd, Belgium).^25^ In the 6 MV dual arc VMAT plans, no robust optimization was employed, and instead a more traditional 3 mm CTV to planning target volume (PTV) margin was employed to ensure sufficient target coverage.

The IMPT plans employed 4 or 5 gantry angles at 45°, 160°, 200°, and 315°; and for some patients an ipsilateral field at 90° or 270°. Gantry angles varied slightly depending on patient geometry. To allow for spot placement in shallow parts of the target, a 4 cm water equivalent path length range shifter was employed at each gantry angle. At 160° and 200° additional fields without range shifters were employed to benefit from smaller spot size at greater depth, resulting in a total of 6 or 7 fields delivered for each patient. In addition to robust optimization, multiple strategies to improve inter‐fraction robustness were employed, such as the avoidance of dose delivery within 8 mm of high‐density dental fillings, which can cause increased density uncertainty, as well as avoidance of beam delivery through the shoulders and skinfold in the neck, which are known for increased positioning uncertainty.^22^ Weekly repeated CT images were made to evaluate the impact of anatomical changes on the treatment. During the clinical IMPT treatment of the six patients, no adaptive replanning for anatomical changes were performed.

### 2.2 PAT treatment planning

Three PAT treatment planning strategies, called ELF,^20^, ^21^ SPArc with energy layer sequencing (SPArc)^18^, ^26^ and early energy layer and spot assignment (ELSA),^19^ were employed to generate seven PAT plans in total for each patient. Plans generated with the ELF and ELSA algorithm were robustly optimized in a research version 12B Alpha of the TPS with GPU accelerated dose computation,^15^ while the plans generated with the SPArc algorithm were robustly optimized in a clinical version 6 of the TPS through scripting as an in‐house developed platform.^26^ The SPArc, ELF and ELSA algorithm were chosen for the comparison, because they were previously published and may soon be available to the radiotherapy community for use in clinical practice. ELF has been implemented in a clinical version (2023B, June 2023) of the TPS and is now used in Trento to produce clinical plans.^27^

Plans using 360° co‐planar gantry rotation with approximately 360 and 240 energy layers were produced using each strategy and optimized to adhere to the clinical treatment planning goals described in Appendix A Table A1. The plans employing 360 energy layers were created to have minimal toxicity, whereas the plans employing 240 energy layers represented a trade‐off between delivery time and toxicity^7^ The ELF plans employed 30 and 10 gantry angles in the plans with 360 and 240 energy layers, respectively, and were spaced equidistantly over the 360° arc. An additional set of ELF plans was produced in which two anterior oblique fields at 36° and 324° employing a range shifter with fully retracted snout and 30 energy layers each were added in an attempt to improve robust target coverage, resulting in a total of 420 energy layers.

**FIGURE 1:**
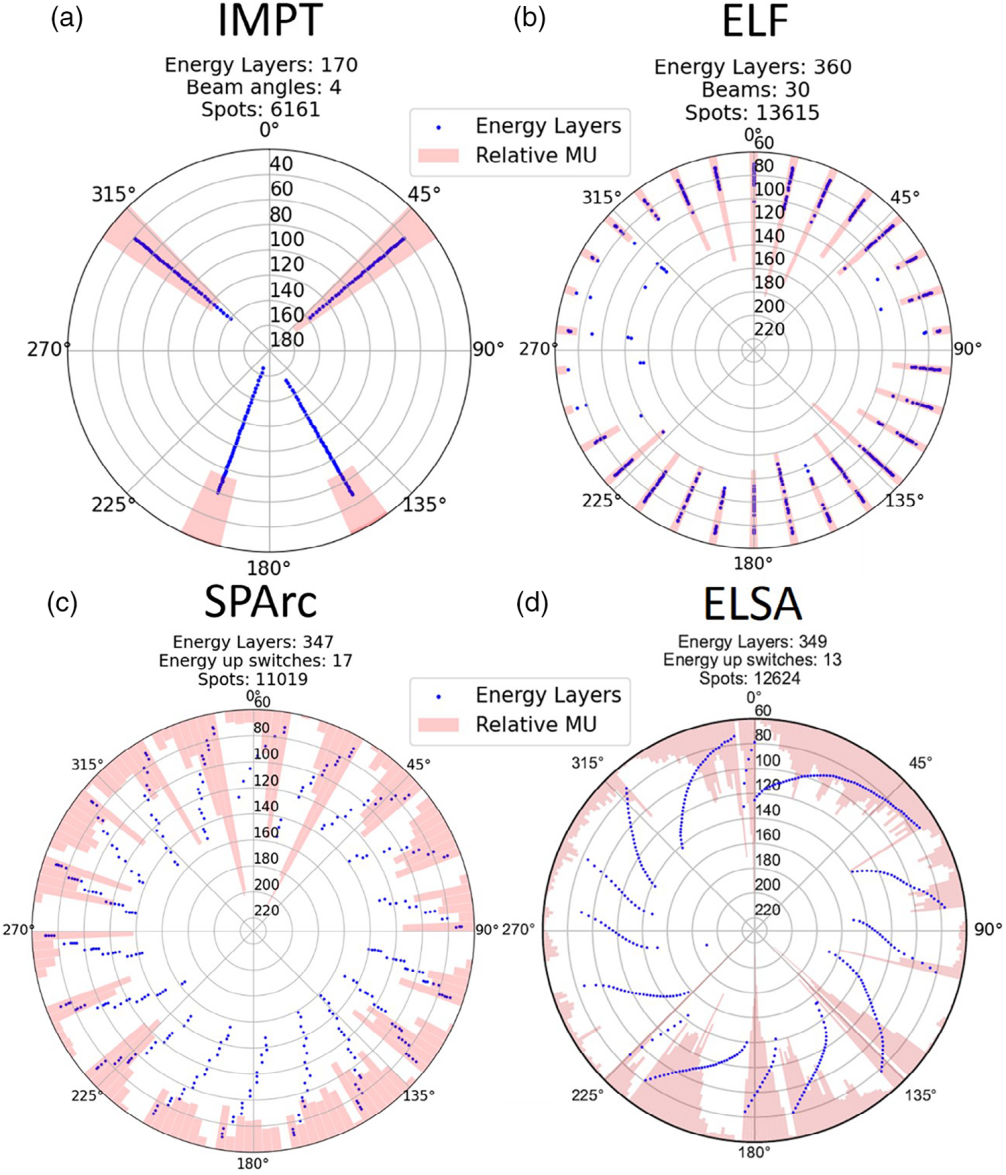
Proton energy in (MeV) and relative MU summed per gantry angle for energy layers distributed over the gantry angles in IMPT (a), and PAT plans produced with ELF (b), SPArc (c), and ELSA (d) algorithms for an OPC patient. ELF, energy layer filtration; ELSA, early layer and spot assignment; IMPT, intensity modulated proton therapy; MU, monitor units; OPC, oropharyngeal cancer; PAT, proton arc treatment; SPArc, spot scanning proton arc.

In the ELF and SPArc optimizations, the number and location of proton energy up‐switches was the same for all patients, depending on algorithm settings employed^7^, ^18^ Whereas in the ELSA optimization, the distribution of energy layers over the arc is optimized based on patient geometry combined with a term to limit the number of proton energy up‐switches^19^ Therefore, the resulting ELSA plans may have varying number‐ and locations of proton energy up‐switches from patient to patient, shown in Figure 1.

The ELF algorithm reduced the initial number of energy layers by half resulting in plans with multiple energy layers per gantry angle suitable for static delivery. The SPArc and ELSA algorithms produced plans with a low number of energy layers at each gantry angle, suitable for delivery with dynamic gantry movement during dose delivery. The SPArc plans used a gantry angle window of 2.5°, over which 1–5 energy layers are delivered, whereas the ELSA plans employed a gantry angle window of 1° in the 360 energy layer plans or 1.5° in the 240 layer plans, over which one single energy layer is delivered. An example of the beam setup in the seven PAT‐, IMPT‐ and VMAT planning approaches is shown in the Supplementary Materials.

### 2.3 Delivery time

Clinical IMPT delivery times were retrieved for 7 fractions per patient, one per treatment week, from the oncology management system (MOSAIQ v2.83, Elekta, California). A model for beam delivery time without gantry dynamics, characterized by the spot delivery time, spot switching time, and energy layer switching time was calibrated using experimental delivery log files from an IBA Proteus PLUS (IBA Ltd, Belgium) treatment delivery system based on the recent published method.^28^ Automated beam sequencing delivery time, in which no user interactions are needed for the delivery of subsequent static beams for ELF and IMPT plan delivery and dynamic delivery time for ELSA and SPArc plans, in which the gantry continuously rotates during dose delivery, were computed using delivery sytem parameters provided by IBA: 0.6°/s^2^ acceleration and 6.0°/s maximal rotational velocity combined with the beam delivery time. As the ELSA and SPArc plans consisted of various sampling frequencies, the dynamic proton arc system controller,^29^ was modified to accommodate the various delivery windows. Gantry acceleration and deceleration during dose delivery and control point switching was allowed and no buffer window was applied in the dynamic arc system controller. An additional time of 1 min per range shifter field was added to the auto beam sequencing delivery time of the IMPT plans, to correct for the time needed to insert the range shifter into the field and move the snout close to the patient surface. The ELF plans with range shifter did not employ snout movements, therefore 30 s were added per range shifter field for moving the range shifter in and out of the beam.

### 2.4 Dose & expected toxicity evaluation

In the nominal dose distributions for the VMAT, IMPT, and PAT plans, the mean dose (*D*mean) in OARs in the NTCP models for xerostomia and dysphagia and NTCP for xerostomia and dysphagia were evaluated^4^ The integral dose was computed by multiplying the *D*mean in the body structure with the volume of the body structure. The maximum dose in 1cc (D1cc) of the body and the maximum dose in 1% volume (D1) of the spinal cord and brainstem were evaluated. To further investigate dose distribution characteristics, we evaluated the conformity index (CI) defined as:

(1) $$\mathrm{CI}=\mathrm{TV}D_{p}/VD_{p}$$

where *TVD_p_* and *VD_p_* are the clinical target volume and total volume covered by the prescribed isodose, and homogeneity index (HI) defined as:

(2) $$\mathrm{HI}=D_{95}/D_{5}$$

where *D_x_* is the minimum dose at *x*% volume of the target.

### 2.5 Inter‐fraction robustness evaluation

Robust target coverage was evaluated on the weekly repeated CT images using 1 mm setup and 3% density uncertainty settings to generate 28 scenarios for evaluation. A smaller setup uncertainty was used in robust evaluation on repeated CTs compared to the setting for robust treatment planning. Patient positioning and anatomical changes are captured in the repeat CT. 1 mm is used for the residual uncertainties, which include the coincidence between the treatment and imaging isocenters, the accuracy of the robotic table correction and the potential difference in rigid registration performed on the rCT in the TPS compared to the CBCT at the treatment gantry.^22^ The maximum dose in 98% of the target volume in a voxel‐wise minimum dose distribution (*D*_98, vwmin28_) constructed from the combined robust scenarios was evaluated.^25^ Furthermore, a cumulative robustness evaluation was performed by warping and accumulating the robust scenarios for each rCT to the planning CT using deformable registration. The cumulative voxel‐wise minimum dose distribution was constructed from the accumulated scenarios, and *D*_98, vwmin_ was evaluated.

## 3 RESULTS

### 3.1 Treatment planning & delivery time

Figure 1 shows for an example patient, the distribution of energy layers over the gantry angles in an IMPT plan and PAT plans employing approximately 360 energy layers produced with the different algorithms. The ELF and IMPT plan employ multiple proton energies at each gantry angle, making them suitable for delivery with a static gantry position. The SPArc and ELSA plans employ a low number or single energy layer per gantry angle, respectively, making them suitable for dynamic gantry rotation during dose delivery. Table 1 shows averaged plan statistics for the different planning strategies employed for six OPC patients.

**TABLE 1:** Average plan statistics for VMAT, IMPT, and proton arc plans generated with the ELF, ELSA, and SPArc algorithms for six OPC patients.

	VMAT	IMPT	10B 240L ELF	240L ELSA	240L SPArc	30B 360L ELF	360L ELSA	360L SPArc	30B 420L ELF + 2RS
Arcs & Fields	2 & N/A	N/A & 6.3	1 & 10	1 & N/A	1 & N/A	1 & 30	1 & N/A	1 & N/A	1 & 32
Gantry angles & control points	180 & 360	4.6 & N/A	10 & N/A	233 & 233	123 & 123	30 & N/A	341 & 341	124 & 124	30 & N/A
Gantry angle window (Deg)	2.0	N/A	N/A	1.5	2.5	N/A	1.0	2.5	N/A
Energy layers	N/A	194	240	233	241	360	341	346	420
Spots 10^3^	N/A	9.1 ± 1.8	11.7 ± 3.9	10.0 ± 2.5	10.8 ± 3.3	15.7 ± 5.3	11.1 ± 3.4	13.7 ± 4.4	17.6 ± 5.6
Energy up‐switches	N/A	5.3 ± 0.5	9.0 ± 0.0	13.0 ± 1.3	17.0 ± 0.0	29.0 ± 0.0	10.7 ± 2.5	17.0 ± 0.0	31.0 ± 0.0
Optimization time (hours)	0.5	0.5	1.2	1.5	6.3^a^(5.5)^b^	1.4	1.9	5.1^a^(4.4)^b^	1.6
Delivery time excluding gantry motion (min)	N/A	4.3 ± 0.4	5.2 ± 0.5	5.7 ± 0.4	5.5 ± 0.5	7.8 ± 1.0	7.4 ± 0.6	7.4 ± 0.7	8.6 ± 1.0
Delivery time (min)		9.3 ± 0.4	6.8 ± 0.5	5.8 ± 0.4	6.2 ± 0.5	9.9 ± 1.0	7.6 ± 0.5	8.2 ± 0.5	11,8 ± 1.0

*Note*: Optimization time was evaluated for a single patient.

Abbreviations: ELF, energy layer filtration; ELSA, early layer and spot assignment; IMPT, intensity modulated proton therapy; OPC, oropharyngeal cancer; PAT, proton arc treatment; SPArc, spot scanning proton arc; TPS, treatment planning system; VMAT, volumetric modulated arc therapy.

^a^ The SPArc algorithm ran on different hardware and used the scripting interface of the older version of the TPS, in which dose computations are not accelerated using a GPU.

^b^ The time for running an in‐house python script for energy layer and spot delivery sequencing and dose re‐calculation.

Optimization time was shortest for IMPT and VMAT plans, followed by plans optimized using the ELF and ELSA algorithms, while the SPArc algorithm took the longest. The ELF and ELSA plans were optimized using an Intel Xeon Gold 6248R CPU and two NVDIA Quadro RTX8000 GPUs.Spot filtering with a minimum threshold of 0.02 MU was performed after 150 iterations of spot weight optimization, after which another 150 iterations were used to reach a stable plan. Similar settings were also employed to estimate optimization time for the clinical IMPT and VMAT plan. The SPArc algorithm was run on different hardware and was not integrated into the TPS, but used the python scripting interface. Most time was spent on running the external script and dose computations which were not accelerated using a GPU.

**FIGURE 2:**
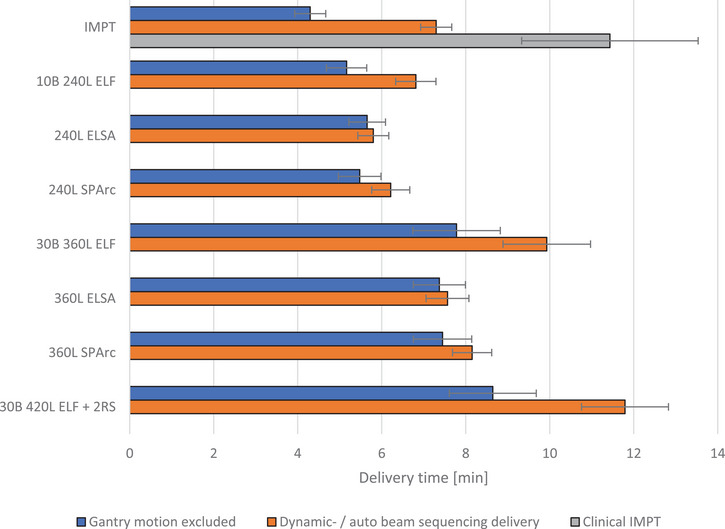
Delivery time for IMPT and PAT plans generated with the ELF, ELSA, and SPArc optimization algorithms for six OPC patients. Delivery time has been estimated for delivery of the fields excluding gantry motion, with gantry motion using auto beam sequencing for IMPT and ELF plans, and with dynamic gantry motion during delivery for the SPArc and ELSA plans. The bars indicate the mean, and the whiskers one standard deviation in the delivery time. ELF, energy layer filtration; ELSA, early layer and spot assignment; IMPT, intensity modulated proton therapy; OPC, oropharyngeal cancer; PAT, proton arc treatment; SPArc, spot scanning proton arc.

Figure 2 shows that, compared to the average clinical IMPT delivery time of 11.4 ± 2.1 min, the delivery time for both dynamic and automated static PAT strategies were shorter, with the shortest delivery time of on average 5.8 ± 0.4 min found for dynamic delivery of the ELSA plans containing 240 energy layers. The longest PAT delivery time with 11.8 ± 1.0 min was found for the ELF plans employing 30 beams, 420 energy layers, and two fields with range shifter.

### 3.2 Dose & expected toxicity evaluation

Table 2 shows dose and toxicity metrics for the plans produced for the six OPC patients. CTV7000 and CTV5425 coverage, *V*_95,vwmin_, was similar between all proton plans. D1 in the brainstem and spinal cord were reduced from on average 28.8 Gy and 41.8 Gy in VMAT to 8.8 Gy and 33.1 Gy in IMPT and to 2.2 Gy and 5.4 Gy in the 360 layer SPArc plans and lower in the other PAT plans, respectively. Compared to VMAT, the average integral dose was reduced by 46% in IMPT and by up to 60% in the PAT plans generated with the ELF algorithm. Compared to IMPT, patient averaged Dmean reduced in all evaluated OARs for each PAT planning strategies. The largest reductions in Dmean were observed in the 360 energy layers ELF plans in the Pharyngeal Constrictor Muscle (PCM) medius, PCM inferior and contra lateral submandibular gland by on average 11.0 Gy, 10.4 Gy and 9.8 Gy, respectively. Dose distributions for an example patient for the various treatment plans is shown in the Supplementary Materials.

**TABLE 2:** Patient averaged dose and toxicity statistics for VMAT, IMPT, and proton arc plans generated with the ELF, ELSA, and SPArc optimization algorithms for six OPC patients.

	VMAT mean ± SD	IMPT mean ± SD	10B 240L ELF mean ± SD	240L ELSA mean ± SD	240L SPArc mean ± SD	30B 360L ELF mean ± SD	360L ELSA mean ± SD	360L SPArc mean ± SD	30B 420L ELF + 2RS mean ± SD
CTV7000 V95, vwmin (%)		98.1 ± 0.7	98.2 ± 0.1	98.1 ± 0.1	98.3 ± 0.2	98.2 ± 0.2	98 ± 0.1	98.2 ± 0.2	98.5 ± 0.2
CTV5425 V95, vwmin (%)		98.4 ± 0.3	98.1 ± 0.1	98.1 ± 0.1	98.1 ± 0.1	98.2 ± 0.1	98.1 ± 0.1	98.2 ± 0.2	98.5 ± 0.3
CTV7000 D98, vwmin28 (%Dp)		94.7 ± 0.6	94.9 ± 0.2	94.7 ± 0.1	94.8 ± 0.1	94.9 ± 0.2	94.8 ± 0.2	94.9 ± 0.2	95.4 ± 0.1
CTV5425 D98, vwmin28 (%Dp)		95.0 ± 0.5	94.7 ± 0.2	94.7 ± 0.2	94.9 ± 0.2	94.8 ± 0.1	94.6 ± 0.1	94.9 ± 0.2	95.6 ± 0.3
CI CTV7000		0.63 ± 0.043	0.68 ± 0.044	0.69 ± 0.035	0.66 ± 0.019	0.67 ± 0.044	0.66 ± 0.081	0.65 ± 0.016	0.67 ± 0.036
CI CTV5425		0.58 ± 0.064	0.62 ± 0.034	0.61 ± 0.041	0.61 ± 0.034	0.65 ± 0.037	0.6 ± 0.047	0.62 ± 0.028	0.64 ± 0.034
HI CTV7000		0.95 ± 0.008	0.97 ± 0.004	0.97 ± 0.005	0.96 ± 0.006	0.96 ± 0.01	0.97 ± 0.005	0.97 ± 0.005	0.97 ± 0.004
HI CTV5425		0.75 ± 0.006	0.76 ± 0.004	0.77 ± 0.005	0.77 ± 0.012	0.76 ± 0.008	0.76 ± 0.008	0.76 ± 0.004	0.76 ± 0
Brainstem D1 (Gy)	28.8 ± 6.3	8.8 ± 5	0.5 ± 0.5	1.2 ± 1.4	1.4 ± 1.8	1.0 ± 1.1	1.0 ± 1.4	2.2 ± 2.9	0.9 ± 0.9
Spinal Cord D1 (Gy)	41.8 ± 3.3	33.1 ± 6.2	4.4 ± 3.5	5.1 ± 4.1	4.5 ± 4.5	4.0 ± 4.2	5.4 ± 4.4	5.4 ± 3.9	3.9 ± 3.4
Body D1cc (Gy)	73.4 ± 0.5	73.9 ± 0.6	73.0 ± 0.2	72.8 ± 0.3	73.2 ± 0.3	72.9 ± 0.2	72.9 ± 0.3	73.1 ± 0.3	73.1 ± 0.1
Integral dose (Gy*L)	171 ± 17	93 ± 9	78 ± 11	84 ± 11	79 ± 10	70 ± 16	83 ± 11	78 ± 9	75.9 ± 10.6
Oral Cavity Dmean (Gy)	42.6 ± 10.1	30.7 ± 12.6	28.8 ± 13.8	29.1 ± 13.8	28.5 ± 13.6	28 ± 13.5	28.9 ± 13.8	28.3 ± 13.5	28.2 ± 13.5
PCM superior Dmean (Gy)	52.8 ± 3.9	44.1 ± 3.7	39.2 ± 6.3	39.5 ± 6.4	38.8 ± 6.1	38.1 ± 6.2	39.3 ± 6.4	38.6 ± 6.0	38.3 ± 6.2
PCM medius Dmean (Gy)	43.9 ± 6.8	34.9 ± 9.8	24.6 ± 13.0	26.5 ± 13.0	24.2 ± 13.2	23.8 ± 12.8	25.7 ± 12.8	24.1 ± 13.0	24 ± 12.8
PCM inferior Dmean (Gy)	27.8 ± 3.3	17.9 ± 2.0	8.2 ± 3.8	10.0 ± 3.4	7.9 ± 2.9	7.5 ± 3.0	9.9 ± 3.9	7.7 ± 3	7.6 ± 3
Cont. Lat. Parotid Dmean (Gy)	18.0 ± 8.2	12.6 ± 6.7	8.9 ± 5.9	9.1 ± 6.1	8.5 ± 6.1	8.4 ± 5.9	9.0 ± 6.0	8.5 ± 5.9	8.5 ± 5.9
Ips. Lat. Parotid Dmean (Gy)	30.7 ± 16.5	27.8 ± 17.6	24.4 ± 19.0	25 ± 18.7	24.2 ± 19.2	23.9 ± 19.1	25.1 ± 18.8	24.1 ± 19.2	24 ± 19.1
Cont. Lat. Submand. gland Dmean (Gy)	44.0 ± 9.7	37.8 ± 13.4	28.0 ± 14.8	24.5 ± 7.2	28.1 ± 16.6	28 ± 16.9	29.5 ± 16.5	28.1 ± 16.7	28 ± 16.8
Ips. Lat. Submand. gland Dmean (Gy)	62.4 ± 6.0	60.9 ± 8.7	55.1 ± 11.4	58.1 ± 10.7	56.5 ± 12.0	55.1 ± 12.4	57.1 ± 12.1	56.3 ± 12.0	56.2 ± 12.4
NTCP Dysphagia gr 2+ (%)	20.6 ± 5.2	11.1 ± 4.3	8.1 ± 4.3	8.4 ± 4.2	7.8 ± 4	7.5 ± 3.8	8.3 ± 4.3	7.7 ± 3.9	7.7 ± 3.10
NTCP Xerostomia gr 2+ (%)	49.0 ± 8.2	45.0 ± 10.4	39.9 ± 10.3	40.4 ± 10.2	39.4 ± 10.2	39.2 ± 10.2	40.3 ± 10.1	39.4 ± 10.2	39.4 ± 10.0
NTCP Dysphagia gr 3+ (%)	4.3 ± 1.1	1.6 ± 0.8	0.9 ± 0.7	0.9 ± 0.7	0.8 ± 0.6	0.8 ± 0.6	0.9 ± 0.7	0.8 ± 0.6	0.8 ± 0.7
NTCP Xerostomia gr 3+ (%)	14.5 ± 3.5	13 ± 4.1	11.1 ± 3.6	11.3 ± 3.6	10.9 ± 3.5	10.8 ± 3.5	11.2 ± 3.6	10.9 ± 3.5	10.9 ± 3.5

Abbreviations: ELF, energy layer filtration; ELSA, early layer and spot assignment; IMPT, intensity modulated proton therapy; NTCP, normal tissue complication probability; OPC, oropharyngeal cancer; PCM, Pharyngeal Constrictor Muscle; SPArc, spot scanning proton arc.

**FIGURE 3:**
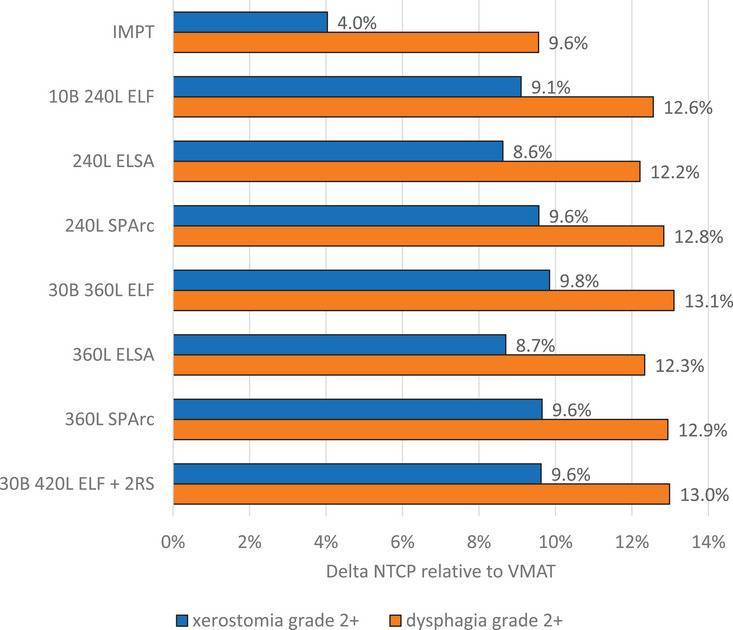
Patient averaged NTCP reduction relative to VMAT for xerostomia and dysphagia grade 2+ for IMPT and PAT plans generated with the ELF, ELSA, and SPArc optimization algorithms for six OPC patients. ELF, energy layer filtration; ELSA, early layer and spot assignment; IMPT, intensity modulated proton therapy; NTCP, normal tissue complication probability; OPC, oropharyngeal cancer; PAT, proton arc treatment; VMAT, volumetric modulated arc therapy.

Figure 3 shows the patient averaged reduction in NTCP relative to VMAT for xerostomia and dysphagia grade 2+ for the different proton plans. Compared with IMPT and VMAT, all PAT planning approaches were able to significantly (*p*‐value < 0.5) reduce NTCP for xerostomia and dysphagia, a reduction in NTCP for each patient was observed. NTCP for xerostomia grade 2+ was reduced relative to VMAT by on average 4.0% in IMPT, 8.7% in ELSA, 9.6% in SPArc, and 9.8% in ELF plans employing 360 energy layers. While NTCP for dysphagia grade 2+ reduced relative to VMAT by on average 9.6% in IMPT, 12.3% in ELSA, 12.9% in SPArc, and 13.1% in ELF plans employing 360 energy layers. Reducing the number of energy layers from 360 to 240 in PAT plans produced with the ELSA and SPArc algorithms resulted in similar NTCP. Whereas NTCP for xerostomia grade 2+ increased by on average 0.7% in the 10 beam 240 energy layer ELF plans compared to the 30 beam 360 energy layer ELF plans. Adding two anterior oblique fields with range shifter to the 360L ELF plans resulted in a minor increase in NTCP of 0.2% and 0.1% for dysphagia and xerostomia grade 2+, respectively.

### 3.3 Inter‐fraction robustness

**FIGURE 4:**
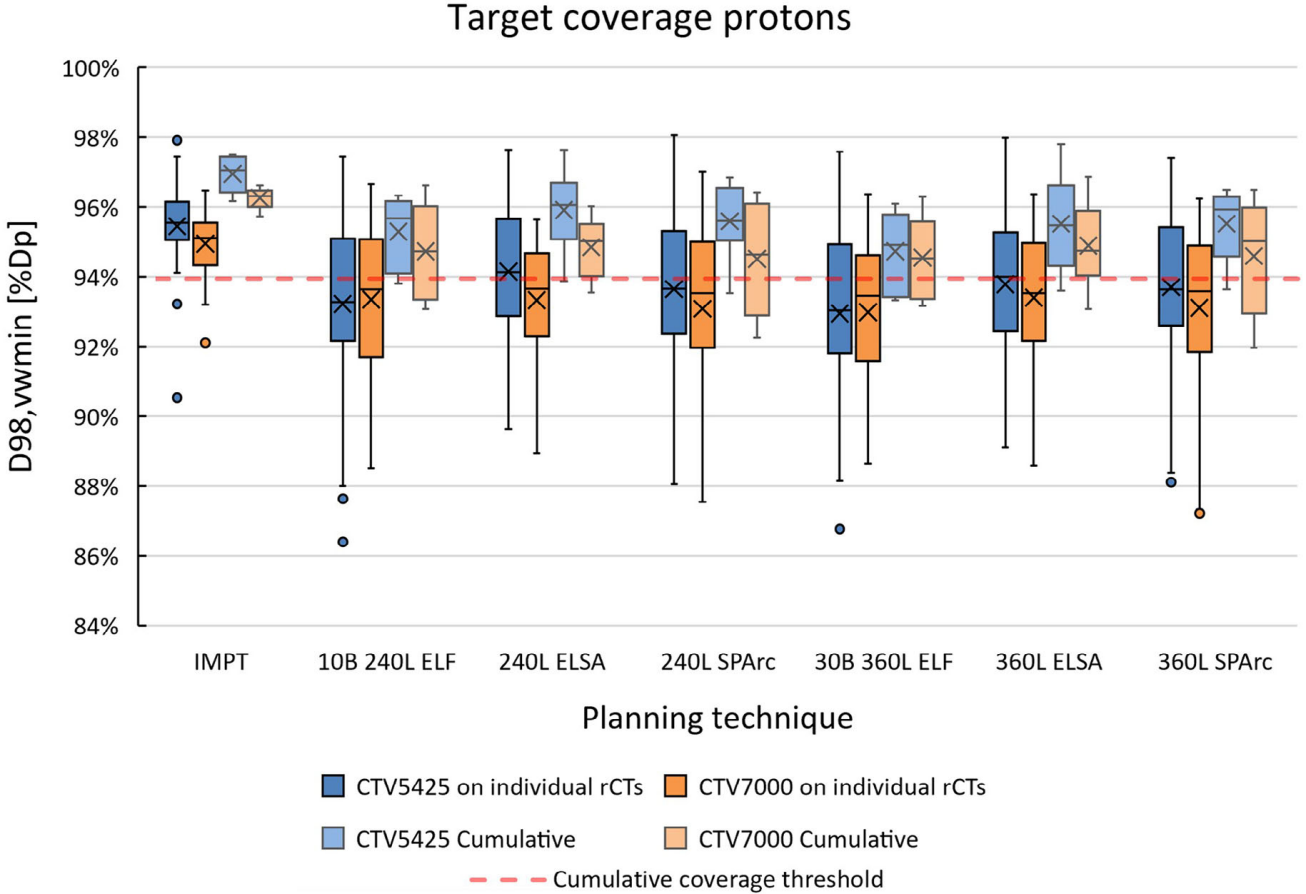
Robust target coverage metrics for IMPT and PAT plans generated using the ELF, ELSA, and SPArc algorithms for six OPC patients. Robust target coverage was evaluated on 42 weekly repeated CTs and accumulated over the repeat CTs for each patient. Dashed line = Cumulative coverage threshold, Box = interquartile range (25th–75th percentile), solid line = median, x = mean, whiskers = range, dots = outliers. ELF, energy layer filtration; ELSA, early layer and spot assignment; IMPT, intensity modulated proton therapy; OPC, oropharyngeal cancer; PAT, proton arc treatment.

Figure 4 shows the robust target coverage on weekly rCTs for all proton plans for the six OPC patients. Robust target coverage on the individual rCTs was better in the IMPT plans with on average a D98, vwmin of 95.0% ± 0.9% prescribed dose (Dp) for CTV7000 and 95.5% ± 1.2% Dp for CTV5425, whereas in the PAT plans without range shifter, on individual rCTs average D98, vwmin ranged between 93.0% ± 2.1% and 93.4% ± 1.8% Dp for CTV7000 and between 92.9% ± 2.5% and 94.2% ± 1.9% Dp for CTV5425. In individual rCT's average D98, vwmin for CTV7000 improved from 93.0% ± 2.1% in the 360L ELF plans to 94.3 ± 1.7 in the 420L ELF plans employing anterior oblique range shifted fields, while average D98, vwmin for CTV5425 improved from 92.9% ± 2.5% in the 360L ELF plans to 94.7 ± 2.2 in the 420L ELF plans employing anterior oblique range shifted fields.

Cumulative target coverage was also better in the IMPT plans with D98, vwmin of on average 96.3% ± 0.3% Dp for CTV7000 and 97.0% ± 0.5% Dp for CTV5425, whereas in the cumulative dose distributions for the PAT planning strategies without range shifter, average D98, vwmin ranged between 94.5% ± 1.6% Dp and 94.9% ± 1.3% Dp for CTV7000 and between 94.7% ± 1.1% Dp, and 95.9% ± 1.2% Dp for CTV5425. Furthermore, it can be noted that the inter‐patient variability in target coverage quantified using D98, vwmin was greater for the PAT strategies compared to IMPT. In the cumulative dose distributions average D98, vwmin for CTV7000 improved from 94.6% ± 1.1% in the 360L ELF plans to 95.4 ± 1.2 in the 420L ELF plans employing anterior oblique range shifted fields, while average D98, vwmin for CTV5425 improved from 94.7% ± 1.1% in the 360L ELF plans to 96.2 ± 1.1 in the 420L ELF plans employing anterior oblique range shifted fields. All the 30B 420L ELF +2RS plans passed our clinical goal for course accumulated target coverage.

## 4 DISCUSSION

The tested early generation PAT planning methodologies showed comparable reductions in dose to healthy tissues and significant NTCP reductions (*p*‐values < 0.05) over clinical IMPT planning. The ELF and SPArc algorithms showed slightly larger toxicity reductions compared to ELSA. Furthermore, each algorithm allowed for generation of PAT plans with reduced estimated delivery time compared to clinical IMPT plans. Compared to SPArc plans, the estimated delivery time for the ELSA plans was slightly lower, most likely caused by the lower number of proton energy up‐switches. However, PAT plans generated by all three algorithms suffer from inter‐fractional robustness problems, which suggests that decreased inter‐fractional robustness maybe an intrinsic property of PAT, rather than a deficiency of a single algorithm. The reduced inter‐fractional robustness of the PAT plans compared to IMPT could in part have been caused by increased conformality of the PAT plans. And inter‐fractional robustness of static PAT plans for OPC patients can be improved by employing specific treatment planning strategies, such as the addition of two anterior oblique range shifter fields. Healthy tissue doses and subsequent NTCP can potentially also be decreased in the clinical IMPT plans by reducing the 3 mm/3% setup/range uncertainty settings used in robust optimization. Reducing the robustness settings has shown a limited reduction in NTCP for grade ≥2 xerostomia on the order of 1.0% per 1 millimeter setup‐ and 0.4% per 1% range uncertainty for IMPT plans.^30^ PAT NTCP could also be further reduced by reducing the uncertainty settings for robust optimization.^31^ Furthermore, the use of range shifters in the IMPT beams could be omitted, which avoids increased spot size caused by proton scatter in the range shifter.^32^

The delivery time estimation showed auto beam sequencing delivery of static PAT plans and delivery of dynamic PAT plans is expected to be faster than current clinical IMPT delivery. IMPT delivery time could be reduced by approximately 2 min when automatic beam sequencing is available. Moreover, expected delivery time for dynamic PAT plans was shorter compared to static PAT plans with the same number of energy layers. Fully automated delivery with synchronized gantry motion and dose delivery is necessary for dynamic PAT delivery, whereas this is not needed for static PAT delivery^7^ This makes static PAT delivery more readily available on current treatment systems. However, when manual inputs are involved between beams in static PAT delivery, delivery time will be extended compared to the reported numbers. The plans generated with the SPArc algorithm contained 1–5 energy layers at each gantry angle and were simulated to be delivered over a gantry angle window of 2.5°. According to the DICOM standard for dynamic PAT delivery, a tolerance gantry angle window should be defined for each energy layer individually. Further processing of the plans generated with the SPArc algorithm can be performed to adhere to the DICOM standard, with potential impact on the dose distribution and delivery time.^33^

Reducing the number of energy layers from 360 to 240 in the dynamic PAT plans resulted in a reduction in delivery time of about 2 min, while having little effect on expected toxicity. There could be potential to reduce the number of energy layers and resulting delivery time in dynamic PAT plans even further without significantly increasing NTCP. Reducing the number of beams from 30 to 10 and the number of energy layers from 360 to 240 in the static PAT plans reduced the delivery time by about 3.5 min. However, the delivery time reduction in the static PAT plan came at the cost of higher expected toxicity, potentially mainly caused by the relatively low number of employed gantry angles.

**FIGURE B1:**
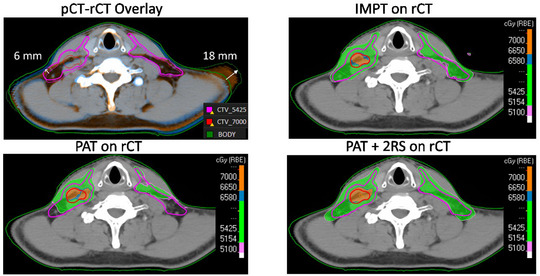
Example of reduced target coverage on weekly repeated CT (rCT) in PAT without range shifter compared to IMPT plan. Top‐left, Difference between planning CT (pCT) and rCT with 18 mm shift in shoulder outline and 6 mm shift of target delineation. V95 and V94 in a voxel‐wise minimum dose distribution on rCT for top‐right IMPT, bottom‐left ELF 30B 360L, bottom right ELF 30B 420 + 2RS plan.

Compared to the IMPT plans, the target coverage in the PAT plans degraded more when recomputed on the weekly repeated CTs. When accumulating the target coverage over the entire treatment, the decrease in D98, vwmin was less severe. Some of the coverage loss was likely caused by patient positioning inaccuracies that are random in nature, the impact of random errors is decreased by averaging over the fractions. In hypo‐fractionated treatments coverage loss caused by random errors could be more problematic. In clinical practice we typically aim for a cumulative CTV coverage of D98, vwmin > 94%. This goal was not met for at least one patient for each PAT planning strategy without range shifter, whereas it was fulfilled by all of the IMPT and ELF plans employing anterior oblique fields with range shifter. In the IMPT plans, strategies are employed to make the dose distributions robust to the most common patient positioning and anatomical changes. For example, to be less sensitive to inaccurate shoulder positioning, shooting through the shoulders is avoided and mostly orthogonal entering beams are selected to reduce the impact of anatomical changes on target coverage. Adding the anterior oblique range‐shifted fields to the ELF plans potentially reduced the lateral contribution of the arc through the shoulders to superficial parts of the target, improving robustness. An example of reduced target coverage in PAT without range shifter compared to IMPT due to shoulder shifts in the rCT compared to the pCT is shown in Appendix B Figure B1. Furthermore, in IMPT treatment adaptive replanning is performed when target coverage evaluated on repeated CTs is lacking because of anatomical changes^22^. Other less common causes for loss of target coverage in the PAT plans included shifts of the patient surface, potentially caused by target shrinkage and/or weight loss. These problems are more systematic in nature and may be better addressed by adaptive replanning.

Decreased inter‐fraction robustness in PAT compared to IMPT could lead to insufficient target coverage or increased adaptive replanning rates, increasing clinical workloads, as discussed in the recent review article on PAT.^34^ Adding anterior oblique range‐shifted fields proved to improve robust target coverage in the ELF plans and limit the need for adaptive replanning. Additional solutions to improve inter‐fraction robustness in PAT similar to ones used in IMPT may be beneficial.^22^, ^24^, ^35^ Alternatively, online‐adaptive replanning methodologies, which have been evolving over recent years, could be employed to ensure target coverage in PAT.^36^ The plans produced with the ELSA algorithm showed slightly better target coverage compared with the other PAT plans, this could have been caused by the dose distributions being somewhat less conformal compared to the other PAT plans. The relation between inter‐fraction robustness and the employed number of energy layers and energy up‐switches in the dynamic PAT plans is not clear from our results, further investigation to establish such a relationship is warranted.^7^, ^37^ The robustness results in this work are specific to PAT for OPC patients, robustness evaluations on rCTs for other treatment sites may yield different results and need further studies.

PAT plan optimization time was more than twice as long compared to the optimization time for IMPT and VMAT. The optimization time for the ELF and ELSA plans could still be acceptable for treatment preparation logistics, whereas the current SPArc plan optimization time would be more cumbersome. However, the SPArc plans were optimized on different hardware and most time in SPArc plan optimization was spend on running the python scripts in the TPS provided interpreter and on CPU based dose computations. These processes can be greatly sped up by proper script integration into a more recent TPS with GPU accelerated dose computation^15^ causing direct comparison of optimization time with SPArc to be of limited value.

The methodologies tested in this study used the proton arc optimization algorithms introduced from 2016 to 2023. Plan optimization time, dose distribution qualities and delivery time are likely to change as the field learns more about all relevant factors.

## 5 CONCLUSION

All three PAT planning methodologies significantly reduced expected toxicity compared to IMPT and VMAT for OPC patients. Estimated PAT deliveries were shorter than clinical IMPT delivery, with dynamic PAT delivery being the fastest, whereas static PAT delivery has less hardware requirements. Reducing the number of energy layers from 360 to 240 in the dynamic PAT plans reduced delivery time and showed limited increase in NTCP. Fraction‐wise robust target coverage was worse in the PAT plans compared to IMPT plans. However, course‐wise robustness was impacted less, and adding anterior oblique range‐shifted fields resulted in acceptable course‐wise target coverage in the ELF static PAT plans.

## CONFLICT OF INTEREST STATEMENT

The authors declare no conflicts of interest.

## Supporting information



Supporting Information

## A Photon and Proton treatment planning goals

**TABLE A1:** Treatment planning goals used for VMAT, IMPT and PAT planning.

	Clinical goal	
Structure	Nominal dose	Voxel‐wise worst dose
CTV7000 (proton plans)		In 16 scenarios V95 > 98% (during planning) In 28 scenarios D98 > 94%Dp (course‐wise)
CTV5425 (proton plans)		In 16 scenarios V95 > 98% (during planning) In 28 scenarios D98 > 94%Dp (course‐wise)
PTV7000 (VMAT)	D98 > 95% Dp	
PTV5425 (VMAT)	D98 > 95% Dp	
Body		D1cc < 78 Gy
Body		D0.03cc < 80 Gy
Eye L&R	D0.1cc < 5 Gy	
Spinal cord, Cerebellum, Cerebrum	D0.1cc < 50 Gy	
Brainstem	D0.1cc < 56 Gy	

Abbreviations: IMPT, intensity modulated proton therapy; PAT, proton arc treatment; VMAT, volumetric modulated arc therapy.
