# Supplementary material for: Early generation dynamic and static proton arc treatment planning algorithms assessment in oropharyngeal cancer patients
Source: Med Phys. 2025 Jul 15;52(7):e17916. doi: 10.1002/mp.17916 (PMC12260774; doi:10.1002/mp.17916)
Supplement: Supplementary file 1 — Supporting Information [file MP-52-0-s001.pdf]

Supplementary materials: Beam setup and dose distributions

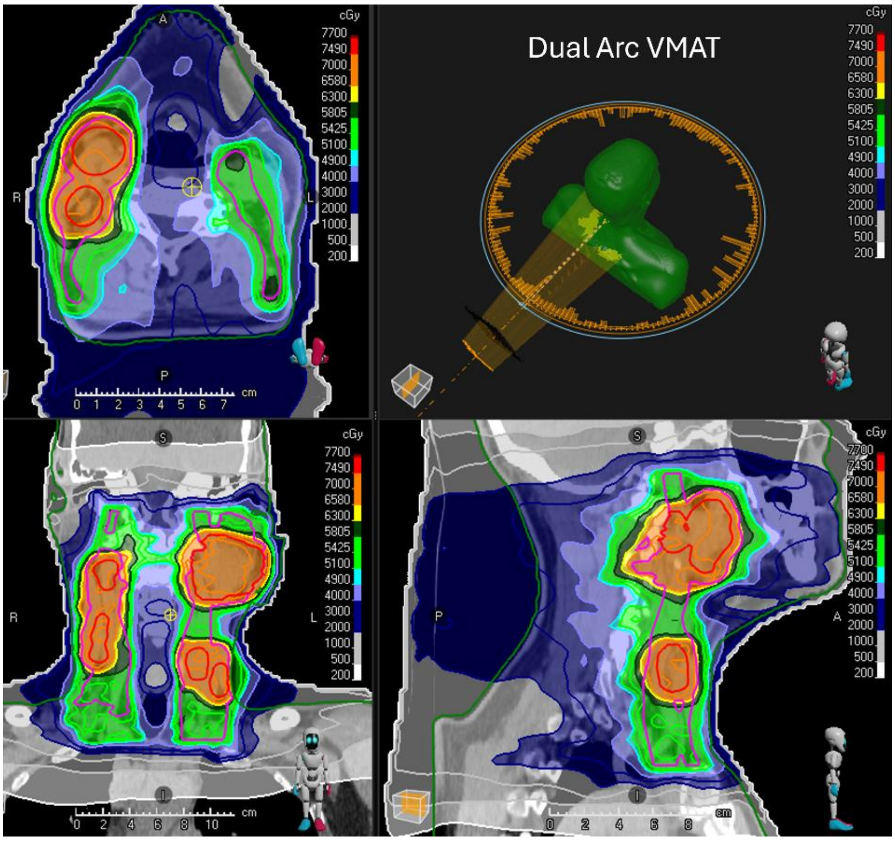

Figure [S6]: Beam setup and dose distributions in the dual arc VMAT plan for an example patient.

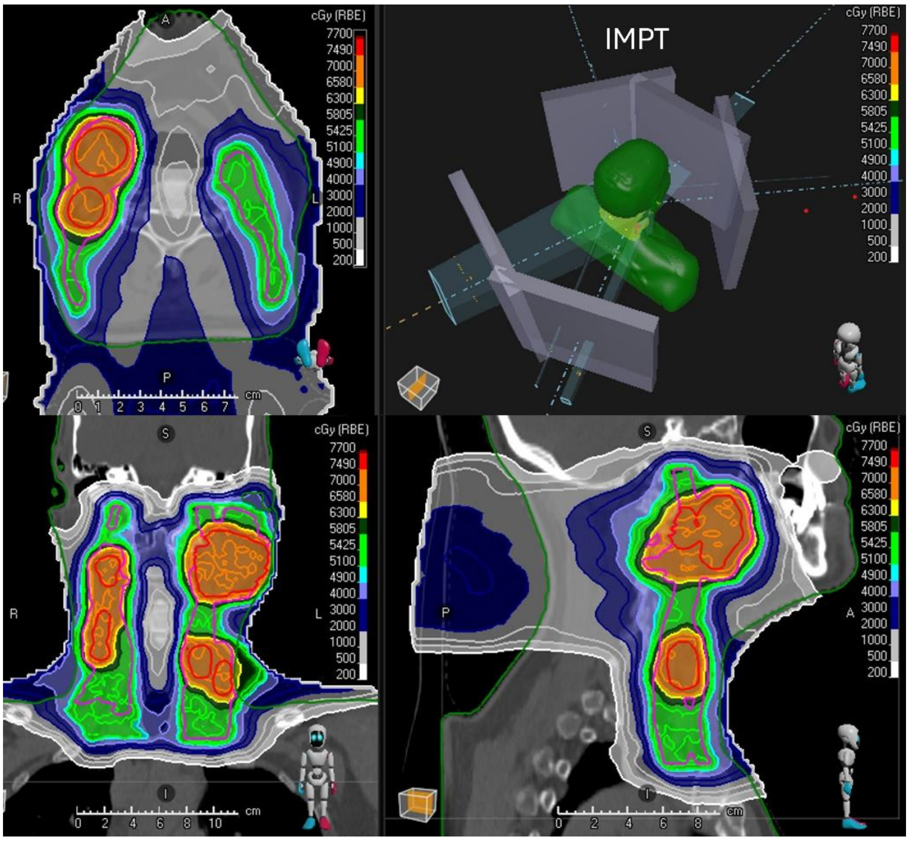

Figure [S7]: Beam setup and dose distributions in the IMPT plan for an example patient, grey blocks indicate range shifters.

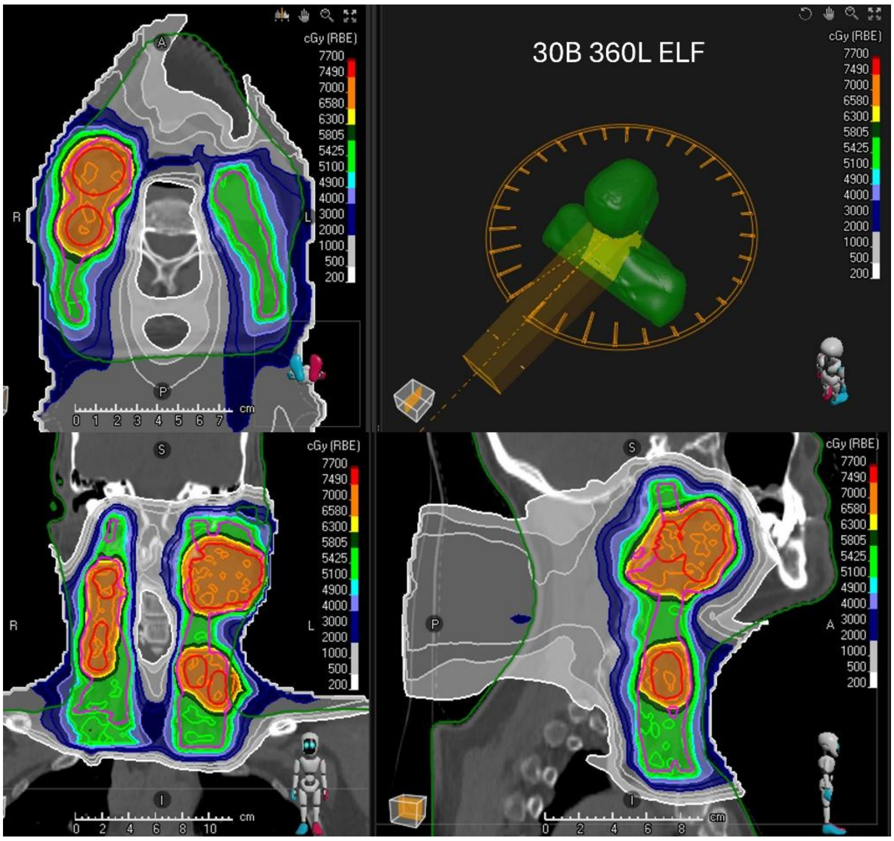

Figure [S8]: Beam setup and dose distributions in the 30B 360L ELF plan for an example patient.

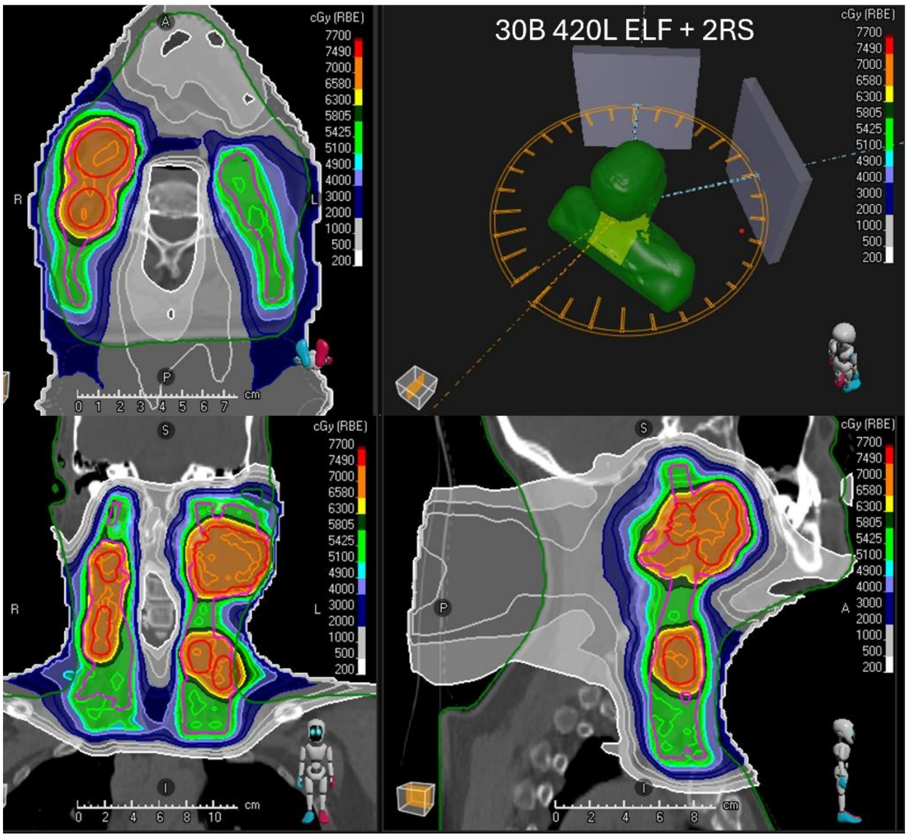

Figure [S9]: Beam setup and dose distributions in the 30B 420L ELF plan for an example patient, grey blocks indicate range shifters.

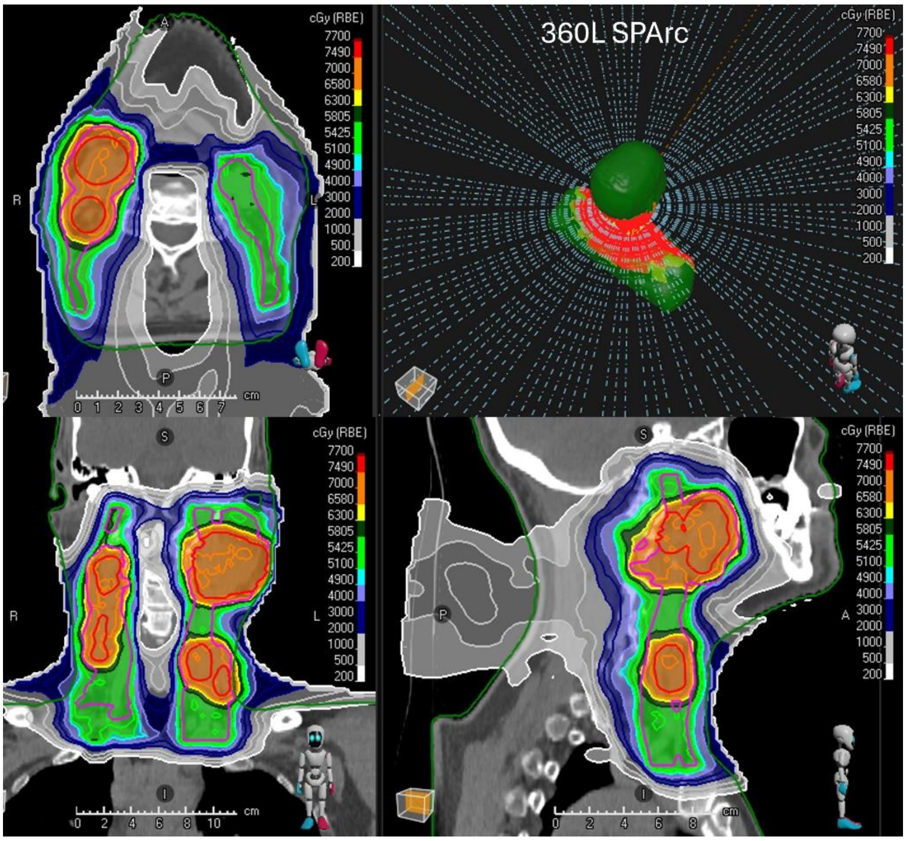

Figure [S10]: Beam setup and dose distributions in the 360L SPArc plan for an example patient.

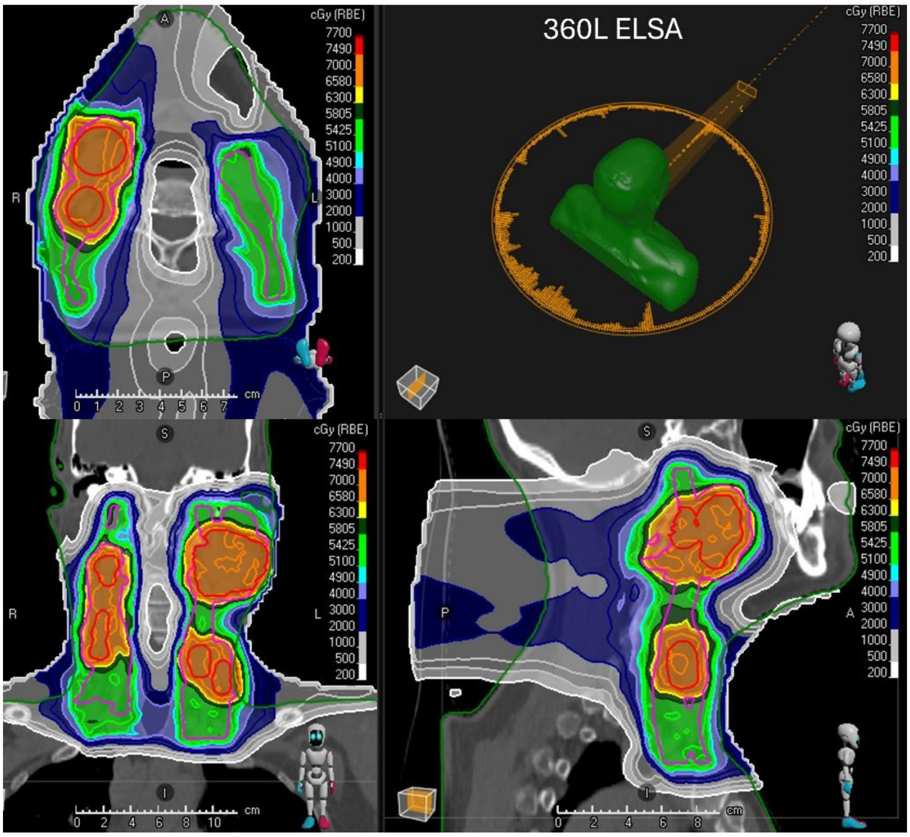

Figure [S11]: Beam setup and dose distributions in the 360L ELSA plan for an example patient.

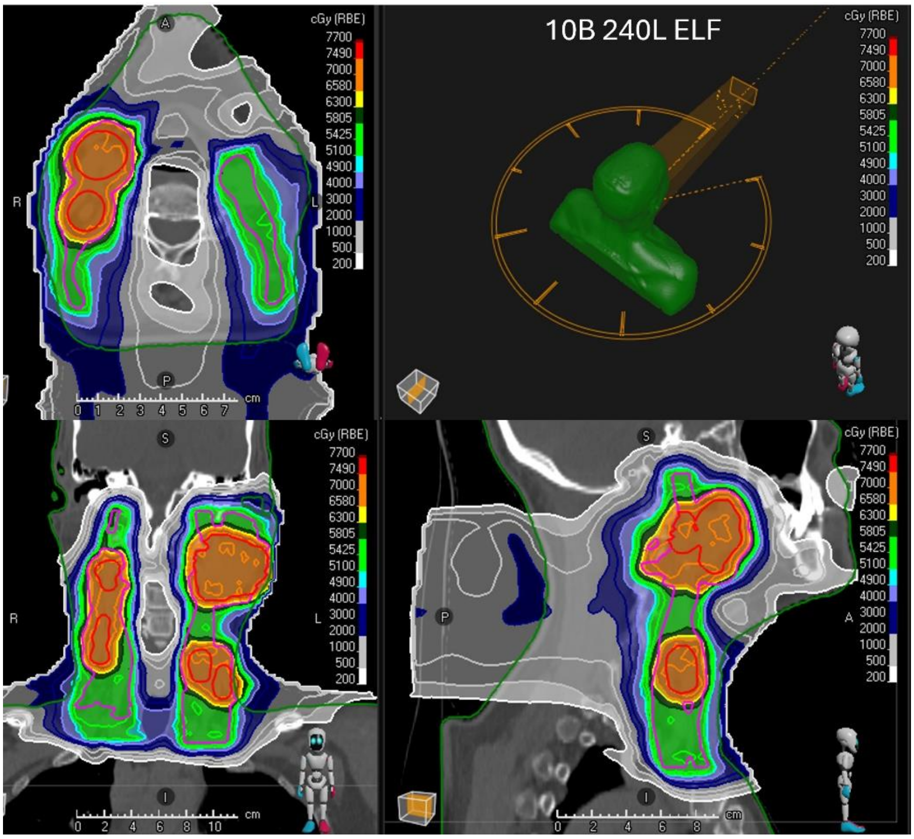

Figure [S12]: Beam setup and dose distributions in the 10B 240L ELF plan for an example patient.

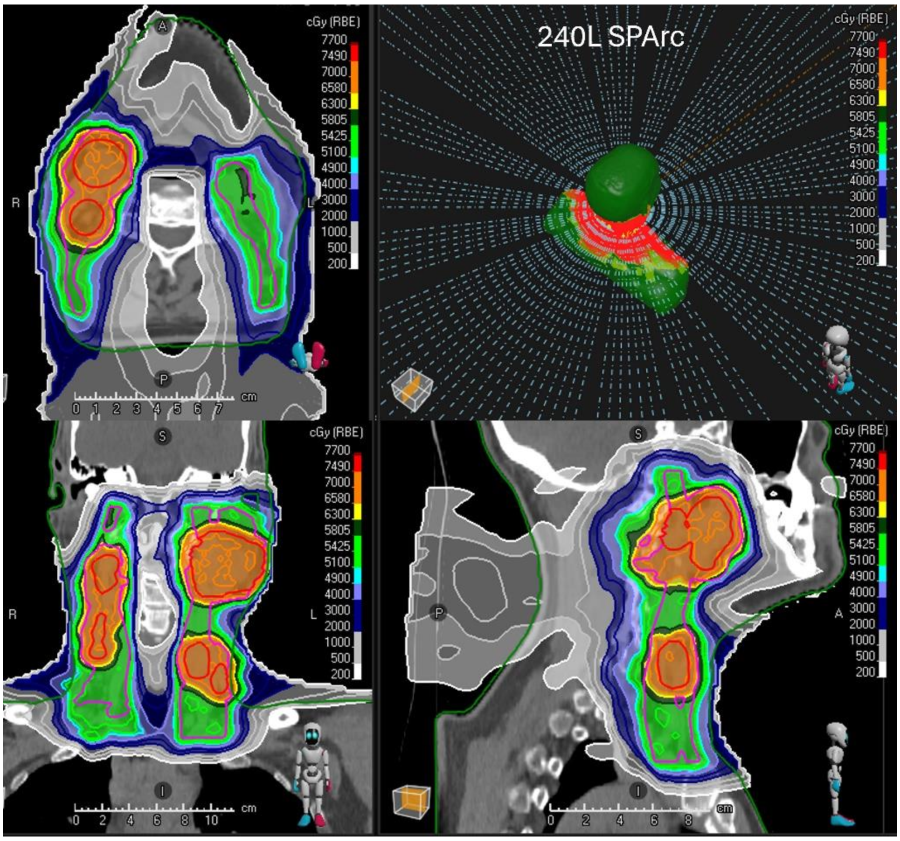

Figure [S13]: Beam setup and dose distributions in the 240L SPArc plan for an example patient.

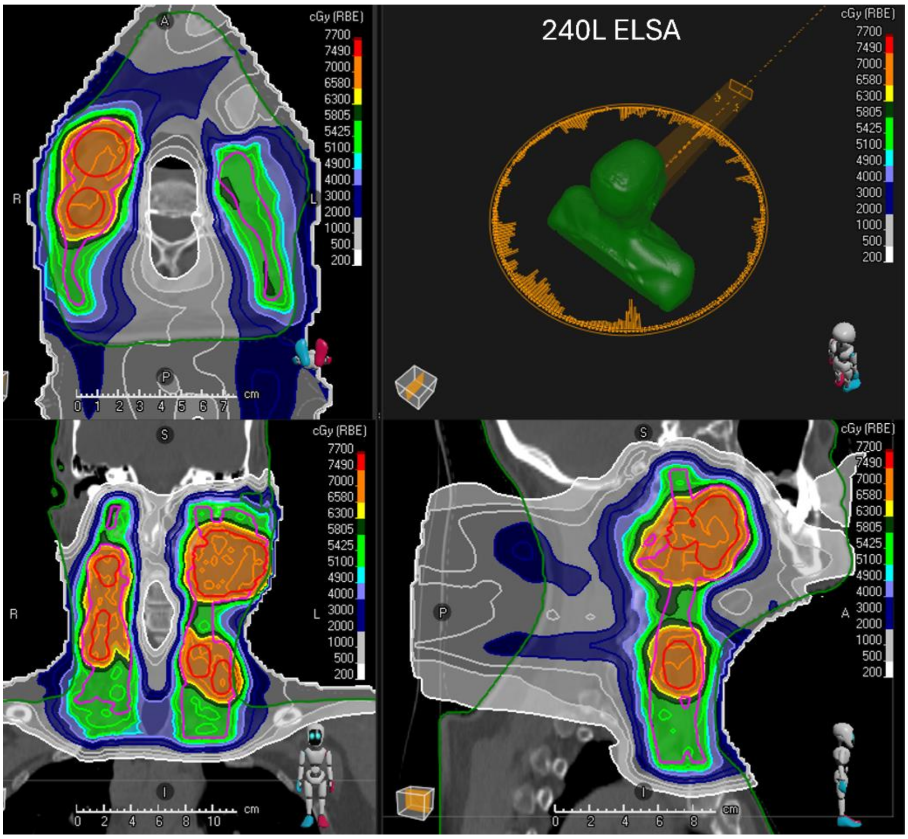

Figure [S14]: Beam setup and dose distributions in the 240L ELSA plan for an example patient.
